# Detection efficiency calibration of single-photon silicon avalanche photodiodes traceable using double attenuator technique

**DOI:** 10.1080/09500340.2015.1021724

**Published:** 2015-03-27

**Authors:** Marco López, Helmuth Hofer, Stefan Kück

**Affiliations:** ^a^ Physikalisch-Technische Bundesanstalt (PTB), Bundesallee 100, 38116Braunschweig, Germany

**Keywords:** detection efficiency, Si-SPAD, photon statistics

## Abstract

A highly accurate method for the determination of the detection efficiency of a silicon single-photon avalanche diode (Si-SPAD) is presented. This method is based on the comparison of the detected count rate of the Si-SPAD compared to the photon rate determined from a calibrated silicon diode using a modified attenuator technique, in which the total attenuation is measured in two attenuation steps. Furthermore, a validation of this two-step method is performed using attenuators of higher transmittance. The setup is a tabletop one, laser-based, and fully automated. The measurement uncertainty components are determined and analyzed in detail. The obtained standard measurement uncertainty is < 0.5%. Main contributions are the transmission of the neutral density filters used as attenuators and the spectral responsivity of the calibrated analog silicon diode. Furthermore, the dependence of the detection efficiency of the Si-SPAD on the mean photon number of the impinging laser radiation with Poissonian statistics is investigated.

## 1. Introduction

Silicon single-photon avalanche diodes (Si-SPADs) gain importance in a variety of different fields of scientific research, i.e. experimental quantum optics, quantum cryptography, and quantum computing, and also in medicine, biology, and astrophysics. Since several years, these diodes are commercially available. The detection efficiency of these devices is one of the most important parameters for application, besides characteristics like detection jitter, dead time, and after-pulsing probability, see for e.g. the overview article about metrology of single-photon sources and detectors by Chunnilall et al. [1].

Measurements of the absolute detection efficiency of photon-counting devices, based on two-photon correlation techniques and on comparison to classically calibrated detectors, have been carried out since several years [2–18]. For the photon correlation technique, a calibrated standard detector is principally not needed, and, thus, also no traceability to an absolute detector is necessary. However, from a metrological point of view, a validation with a standard detector traceable to the cryogenic radiometer or to a calibrated lamp is necessary, because otherwise two independent scales for the optical power would exist. Thus, conventional calibration methods (usually traceable to a cryogenic radiometer or a standard lamp) for validation were carried out, e.g. see Migdall et al. [2] and references therein as well as selected data in Table 1.

**Table 1.** Calibration results for single-photon detector in the visible spectral range based on conventional techniques.

Method	*u* (DE)	Wavelength (nm)	Ref.
Attenuated filtered lamp	20%	668.5 ± 1.5	[3]
Attenuated HeNe-laser	18%	632.8	[4]
Attenuated HeNe-laser	1.7%	632.8	[9]
Attenuated HeNe-laser	1.3%	632.8	[10]
Attenuated HeNe-laser	6.8%	632.8	[13]
Attenuated filtered broadband source	0.17	703 ± 3	[14]
Attenuated filtered broadband source	68%	702 ± 5	[17]
Synchrotron radiation	0.16	651	[18]
Attenuated laser	0.2	770.00 ± 0.06	This work

In the work presented in this paper, a tabletop, easy-to-handle measurement setup and technique for the determination of the detection efficiency of Si-SPADs is presented, including a detailed measurement uncertainty analyses. Full traceability to the national primary standards is assured via an unbroken calibration chain. The detection efficiency is determined from the measurement of the photon count rate of the Si-SPAD and its comparison to the incoming photon flux, which is measured with a calibrated Si photodiode and calibrated attenuators. The standard measurement uncertainty is currently < 0.5%.

For the calibration, a laser source is used; therefore, the Poissonian photon number distribution has to be taken into account in the calibration process, as investigated in the work of Schmunk et al. [19]. Depending on the attenuation, the mean photon number applied to the detector varies in a wide range; thus, in combination with the detector dead time, this leads to a strong dependence of the measured detection efficiency from the incoming photon flux.

## 2. Calibration setup and procedure

In the setup presented in the paper, the calibration of an Si-SPAD (Perkin-Elmer-SPCM-AQR-16) is carried out via comparison with a calibrated Si-diode (Hamamatsu S1227), which acts as the reference detector, using a continuous-wave laser source operating at 770 nm. This comparison is not possible to perform directly, because of the different photon fluxes that need to be applied to the detectors. This is taken care of by using appropriate neutral density filters, which are calibrated *in situ* in the same setup. However, the attenuation of such a filter has to be in the order of 10^7^–10^8^ in order to attenuate the incoming photon flux from the laser down to single-photon level. Such a high attenuation is practically not possible to be measured with a standard Si-diode, because in this case the photon flux is simply too small. To overcome this problem, the following procedure was used and implemented in the setup shown in Figure 1 [Fig F0001b]. The power of a stabilized diode laser operating at a wavelength of 770 nm is directly measured with the standard Si-diode. Then, a filter 2 (NG9, OD5.0, *T* ~ 4.6 × 10^−4^) is moved into the beam and the corresponding power is also measured with the standard Si-diode. The same procedure is repeated for filter 3 (Filter package consisting of NG9, OD2.6, and NG9, OD3.0, total transmittance ~ 1.6 × 10^−4^). For the translation of the neutral density filters, computer-controlled translation stages with an accuracy of 1 μm were used, so that the filters were always irradiated at the same position, thus avoiding effects of filter inhomogeneity. Therefore, highly accurate *in situ* measurements of the filter transmissions of filter 2 and filter 3 are performed. However, the effect of combining two filters, which is necessary for the measurement with the Si-SPAD detector, still has to be taken into account. For the validation of the applicability of the above-described procedure and a discussion of associated uncertainty of the filter transmission, see Section 4. In the final step of the calibration procedure, both filters as well as the Si-SPAD are placed in the beam path. From the known laser power and the measured filter transmissions, the incoming photon flux onto the Si-SPAD is now known, and thus the detection efficiency can be determined. For varying the laser power and thus the photon rate impinging on the detector, an additional variable filter (Thorlabs, NDL-10S-4, with optical densities between 0.1 and 4) was placed right in front of the laser. This filter has no further effect on the measurement other than changing the radiative flux. Because the laser itself could be operated at the same power level, the radiative flux is much more stable than in the case where the radiative flux was changed by adjusting the operational conditions of the laser. It should be noted that both detectors, the standard Si-diode as well as the Si-SPAD, are underfilled by the incoming beam; this is assured by the use a microscope objective (Mitutoyo M Plan Apo, 20x, NA = 0.42), which remains in the beam throughout the whole calibration procedure.

**Figure 1a.**
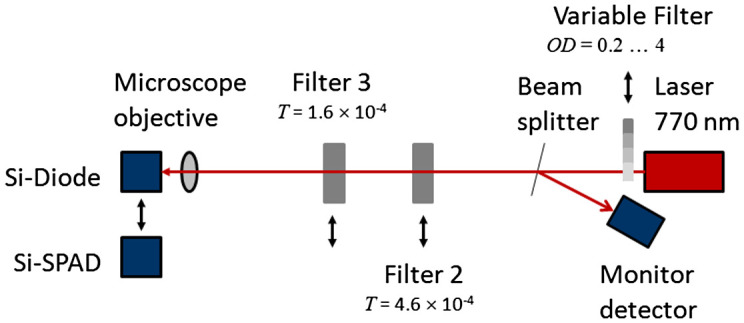
Schematic setup for the detection efficiency calibration of Si-SPADs. (The colour version of this figure is included in the online version of the journal.)

**Figure 1b. F0001b:**
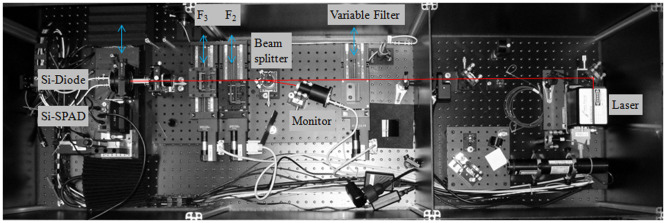
Photograph of setup for Si-SPAD detector calibration. (The colour version of this figure is included in the online version of the journal.)

## 3. Determination of the detection efficiency

In the procedure described above, the following four signals *V*_1_ to *V*_4_ are measured:

(1) $$\begin{array}{c} \left.\begin{array}{c} V_{1}=A_{1}\cdot s_{\text{Si,1}}\cdot \Phi_{1}\\ V_{2}=A_{2}\cdot s_{\text{Si,2}}\cdot \Phi_{2}=A_{2}\cdot s_{\text{Si,2}}\cdot T_{\text{F2}}\cdot \Phi_{1}\\ V_{3}=A_{3}\cdot s_{\text{Si,3}}\cdot \Phi_{3}=A_{3}\cdot s_{\text{Si,3}}\cdot T_{\text{F3}}\cdot \Phi_{1}\\ V_{4}=CR=\eta \cdot \frac{\Phi_{4}}{hc/\lambda }=\eta \cdot \frac{T_{\text{F2}}\cdot T_{\text{F3}}\cdot \Phi_{1}}{hc/\lambda } \end{array}\right\}\\ \Rightarrow \eta =\frac{hc}{\lambda }\cdot \frac{A_{2}\cdot A_{3}\cdot }{A_{1}\cdot }\cdot \frac{CR\cdot V_{1}}{V_{2}\cdot V_{3}}\cdot \frac{s_{\text{Si,2}}\cdot s_{\text{Si,3}}}{s_{\text{Si,1}}} \end{array}$$

With: *η*: detection efficiency, *V*_i_: signals (*V*_1_: signal for the measurement of the laser power, *V*_2_: signal for the measurement of filter 2, *V*_3_: signal for the measurement of filter 3, *V*_4_: signal (= count rate *CR*) for the measurement with the Si-SPAD, *s*_Si,i_: spectral responsivitiy of the Si-diode for the different irradiation levels, *A*_i_: amplification factors of the transimpedance amplifier in the measurements 1–3, Φ_i_: radiant powers, *T*_F2_, *T*_F3_: filter transmissions, *CR*: count rate of the Si-SPAD, *h*: Planck constant, *c*: speed of light, and *λ*: wavelength. It should be noted that the detection efficiency is defined here purely as the number of counts divided by the number of impinging photons, i.e. other effects, as e.g. after-pulsing, are not considered.

This equation is valid only if the laser power does not change during the measurements. This is taken into account by applying the monitor principle in the measurements. This means, that instead of using the signals directly, the ratios *Q*_i_ between the measured signals *V*_i_ and the simultaneously measured monitor signals *V*_mon,i_ are used. Furthermore, the responsivities of the Si-diode is practically linear for the applied power levels between approx. 1 nW and 10 μW (the dynamic range of this type of diode is approx. 3 pW … 300 μW), and thus taken into account by an uncertainty component, see Section 4. So, the following equation for the detection efficiency is finally obtained:

(2) $$\eta =\frac{hc}{\lambda }\frac{A_{2}A_{3}}{A_{1}}\frac{V_{1}/V_{\text{Mon1}}CR/V_{\text{Mon4}}}{V_{2}/V_{\text{Mon2}}V_{3}/V_{\text{Mon3}}}s_{\text{Si}}F_{\text{filt}}=\frac{hc}{\lambda }\frac{A_{2}A_{3}}{A_{1}}\frac{Q_{1}Q_{4}}{Q_{2}Q_{3}}s_{\text{Si}}F_{\text{filt}}$$

With *F*_filt_ = 1 ± *u*(*F*_filt_): uncertainty related to the filter transmission determination, see Section 4.

## 4. Determination of the measurement uncertainty

In Table 2, all components for the determination of the detection efficiency and its measurements uncertainty, based on Equation (2), are listed for the example of a photon rate of approx. 100,000 photons/s. In the following, the different components will be discussed in detail.

**Table 2.** Measurement uncertainty budget.

Measurand	Unit	Description	Value	Standard uncertainty	Distribution	Sensitivity coefficient	Contribution	Contribution (%)
*h*	Js	Planck constant	6.626206957 × 10^−34^ Js	1.67 × 10^−42^ Js	Rectangular	960 × 10^30^	1.6 × 10^−9^	0.0
*c*	m/s	Speed of light	299.792458 × 10^6^ m/s					
*λ*	m	Wavelength	770.0000 × 10^−9^ m	57.7 × 10^−12^ m	Rectangular	−830 × 10^3^	−48 × 10^−6^	0.0
*A*_1_	V/A	Amplification factor	1.0005070 × 10^6^ V/A	20.8 V/A	Rectangular	640 × 10^−12^	13 × 10^−6^	0.0
*A*_2_	V/A	Amplification factor	1.0009520 × 10^9^ V/A	53,100 V/A	Rectangular	640 × 10^−12^	34 × 10^−6^	0.0
*A*_3_	V/A	Amplification factor	1.0009520 × 10^9^ V/A	53,100 V/A	Rectangular	640 × 10^−12^	34 × 10^−6^	0.0
*Q*_1_	1	Ratio *V*_1_/*V*_Mon1_	0.7929810 1	31.7 × 10^−6^ 1	Standard	0.80	25 × 10^−6^	0.0
*Q*_2_	1	Ratio *V*_2_/*V*_Mon2_	0.3642505 1	52.7 × 10^−6^ 1	Standard	−1.7	−92 × 10^−6^	0.2
*Q*_3_	1	Ratio *V*_3_/*V*_Mon3_	0.01229298 1	6.16 × 10^−6^ 1	Standard	−52	−320 × 10^−6^	2.6
*Q*_4_	1/V/s	Ratio *CR*/*V*_MonSPAD_	38973.0 1/V/s	14.1 1/V/s	Standard	16 × 10^−6^	230 × 10^−6^	1.3
*s*_Si_	A/W	Spectral responsivity of Si-Diode	0.357750 A/W	716 × 10^−6^ A/W	Standard	1.8	1.3 × 10^−3^	40.9
*F*_F_	1	Factor for the use of two filters	0.99680 1	2.31 × 10^−3^ 1	Rectangular	0.64	1.5 × 10^−3^	54.8
*η*	1	Detection efficiency	0.63586	1.99 × 10^−3^	Standard			

### 4.1. Constants

*h*: Planck-constant, *h* = 6.62606957 (29) × 10^−34^ Js [20].
*c*: speed of light, *c* = 299,792,458 m/s [21].

### 4.2. Wavelength

*λ*: wavelength, *λ* = 770.00 nm ± 0.06 nm (New Focus, TLB 6312 [22]).

### 4.3. Amplification factors

The amplification factors and their associated uncertainties of the current to voltage amplifier (Gigahertz-Optik, P-9202-5) were determined from the calibration performed at the PTB’s electricity and magnetism division [23].

*A*_1_: amplification factor, *A*_1_ = 1,000,507 V/A ± 21 V/A.
*A*_2_: amplification factor, *A*_2_ = 1,000,952,000 V/A ± 53,100 V/A.
*A*_3_: amplification factor, *A*_3_ = 1,000,952,000 V/A ± 53,100 V/A.

### 4.4. Signal ratios

The signals of the counter and of the monitor detector were taken from the 10 simultaneous readings of a frequency counter (Agilent 53131A) and a digital multimeter (Agilent 34401A). From these 10 ratios, *Q*_4_ ± *u*(*Q*_4_) was determined.

*Q*_4_: ratio *CR*/*V*_MonSPAD_, *Q*_4_ = 38973.0 1/V/s ± 14.1 1/V/s.

The signals of the standard Si-diode and of the monitor detector were taken from the 10 simultaneous readings of two multimeters (Agilent 34401A). From these 10 ratios, *Q*_i_ ± *u*(*Q*_i_) were determined.

*Q*_1_: ratio *V*_1_/*V*_Mon1_ = 0.7929810 ± 31.7 × 10^−6^.
*Q*_2_: ratio *V*_2_/*V*_Mon2_ = 0.3642505 ± 52.7 × 10^−6^.
*Q*_3_: ratio *V*_3_/*V*_Mon3_ = 0.01229298 ± 6.16 × 10^−6^.

### 4.5. Spectral responsivity

**Figure 2.**
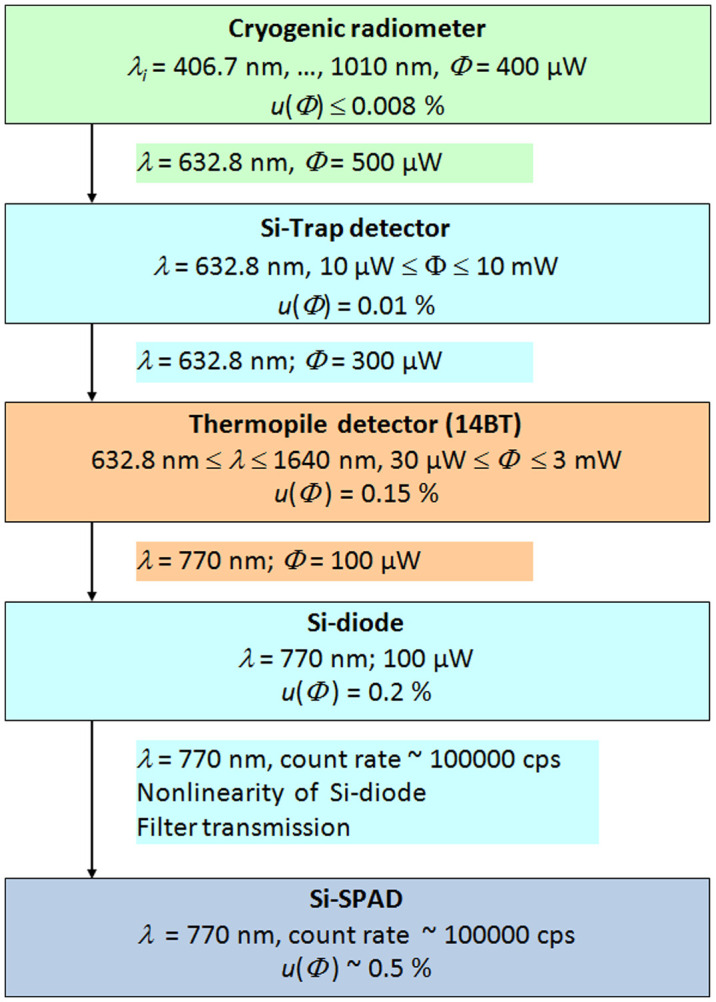
Calibration chain for the determination of the Si-SPAD detection efficiency. (The colour version of this figure is included in the online version of the journal.)

The spectral responsivity of the Si-diode was calibrated against a 14BT thermopile detector at a wavelength of 770 nm. This thermopile detector was calibrated at the Helium–Neon laser wavelength of 633 nm against an Si-trap detector, which was calibrated against the primary standard for optical radiative power, i.e. the cryogenic radiometer [24]. The spectral dependence of the thermopile detectors responsivity is known from its wavelength dependence reflectivity, measured at the Photometry and Applied Radiometry Department of PTB. In Figure 2, the calibration chain for the determination of the detection efficiency of the Si-SPAD detector is shown for overview purposes.

*s*_Si_: spectral responsivity of the Si-diode, *s*_Si_ = 0.357750 A/W ± 716 × 10^−6^ A/W.

### 4.6. Filter transmission

For the determination of the measurement uncertainty of the total filter transmission, a very similar procedure as in the calibration process was applied. However, neutral density filters with lower optical densities were used. Thus, it was possible to determine the filter transmission of each individual filter, and also the overall transmission of the filter combination. For filter 2′ (NG11, OD 0.3), the transmission was *T*_F2′_ = 0.3748 ± 0.0004, for Filter 3′ (NG4, OD 0.6), the transmission was *T*_F3′_ = 0.3302 ± 0.0004. Thus, from the individual measurements, we obtain *T*_single_ = *T*_F2′_ × *T*_F3′_ = 0.1238 ± 0.0002. From the combined measurement, we obtained directly *T*_combined_ = 0.1234 ± 0.0004. Therefore, we have a deviation between the two measurements of 0.3%, resulting in a correction factor *F*_F_ = *T*_combined_/*T*_single_ = 0.9968. The standard uncertainty is estimated as the difference *T*_single_ − *T*_combined_ = 4 × 10^−3^. However, further measurements are necessary in order to verify if the deviation is a constant correction factor or just arises from statistics.

*F*_F_: correction factor that takes into account the combined use of the two filters.

$$F_{\text{F}}:=0.9968\pm 0.0023$$

### 4.7. Total measurement uncertainty

Taking into account all the above-stated components of the measurement uncertainty budget, an overall expanded measurement uncertainty (*k* = 2) for the detection efficiency of Si-SPAD detectors of 0.63 % is obtained. All the details are summarized in Table 2. Besides the measurands and its description, also the unit, the standard measurement uncertainty, the distribution, the sensitivity coefficient, the absolute contribution and the relative contribution to the total uncertainty are given.

Main contribution to the overall uncertainty is, as expected, the uncertainty in the filter transmission, which contributes to about 55% of the total uncertainty. However, in our setup, utilizing *in situ* measurements with computer-controlled stages assure highly reproducible results. Despite that, deviations in the order of a few per mile occur between the individual measurements and the combined measurements, whose origin are not yet clear. Currently investigated are larger distances between the filters and larger bandwidths of the laser sources in order to reduce interference effects from the filter surfaces.

Another large contribution of about 41% arises from the standard Si-detector. This is mainly caused by the uncertainty in the nonlinearity measurement, because the applied fluxes cover about four orders of magnitude. The measurements actually indicate a linear behavior, however, caused by the applied flux addition method; the uncertainty in the measurement itself is rather high and has to be taken into account. This contribution might be reduced by improving the nonlinearity measurements, which will be carried out in the nearest future.

The other components are currently of less importance for the overall uncertainty, so they will not be addressed in the nearest future. It should be noted that the uniformity of an Si-SPAD detector might be an issue for the determination of the detection efficiency. However, in the measurements described here, we were able to realize a high reproducibility, as can be seen from the small uncertainty in the *Q*_4_ value, see Section 4.4 and Table 2.

### 4.8. Calibration result

For photon fluxes of approx. 10^5^ cps, we finally obtain for the detection efficiency of the Si-SPAD investigated:

*η*_SPAD_ = 0.6359 ± 0.0040 (*k* = 2).
*η*_SPAD_ = 0.6359 ± 0.63% (*k* = 2).

The detection efficiency is valid, as usual for calibrations, only for the measurement conditions stated above, for other conditions (e.g. wavelength, photon number distribution, temperature, etc.) the value needs to be corrected. The influence of the total photon flux on the detection efficiency is discussed in the next section.

### 4.9. Photon flux dependence of the detection efficiency – dead time and photon statistics effects

**Figure 3.**
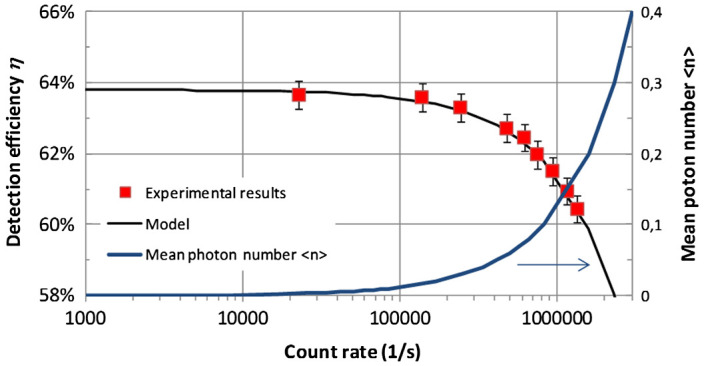
Detection efficiency dependence from the photon count rate. The experimental results (red squares) are best described with *η*_0_ = 63.8% and *t*_dead_ = 75 ns. (The colour version of this figure is included in the online version of the journal.)

An Si-SPAD exhibits a strong dependence of the detection efficiency from the photon number impinging on it. The total number of photons per second detectable by an Si-SPAD detector is limited by the dead time, i.e. the time that the Si-SPAD is blind to a photon, because it has to recover from a previous photon detection event. Typically, the dead times for Si-SPADs are between 10 and 100 ns. Strongly correlated with the dead time is the time distribution of the photons arriving on the Si-SPAD. Photons arriving singly with a time distance larger than the dead time may be completely detected by the Si-SPAD; the same number of photons arriving within one pulse would just allow one detection event. Thus, the photon number distribution of the photon source used in the calibration experiment is highly important [25]. The best light source that can be used for determining the undisturbed detection efficiency of a Si-SPAD would be a single-photon source, which delivers single photons separated with a time distance larger than the dead time of the Si-SPAD and with a photon flux high enough to be measured by means of a conventional Si-diode. However, such single-photon sources are not available to date. Therefore, in the calibration processes against classical Si-photodiodes, mostly laser sources are used. They exhibit a Poissonian photon emission number distribution, which can be easily described with the mean photon number ⟨n⟩. For the investigation of the influence of photon rate, and thus mean photon number, on the detection efficiency of an Si-diode, our setup described above was modified in a way, that an additional variable neutral density filter was placed in the beam path, see Figure 1 [Fig F0001b]. Doing this, we were able to adjust the photon flux rate impinging on the Si-SPAD between 1000 photons per second and 3,000,000 photon per second. In Figure 3, the result of the calibration against a standard Si-diode, which is unaffected by the photon number distribution of the applied source, is shown. As expected, a strong dependence of the detection efficiency of the Si-SPADs as a function of the count rate is clearly observed; for about 100,000 counts per second (corresponding to mean photon numbers of about 0.1), the measured detection efficiency starts to drop significantly. In order to describe the observed behavior, we divided the continuous-wave laser beam into pulses with the length of the dead time of the detector, i.e. into pulses with a repetition rate 1/*t*_dead_. Then, for a Poissonian source, the SPAD count rate and the detection efficiency can be described by:

Count rate at SPAD:

(3) $$CR=\frac{1-\exp\left(-\eta_{0}\langle n\rangle \right)}{t_{\text{dead}}}$$

Detection efficiency:

(4) $$\eta =\frac{1-\exp\left(-\eta_{0}\langle n\rangle \right)}{\langle n\rangle }$$

Fitting the dead time *t*_dead_ and the low signal detection efficiency *η*_0_, the observed behavior of the detection efficiency in dependence of the detector count rate can be described very well, see also Figure 3. It should be noted that for even higher count rates (not shown here) additional effects (like e.g. after pulsing) occur, significantly influencing the detection efficiency.

### 4.10. Measurements of two silicon single-photon avalanche detectors with different detection efficiency

The measurements of the photon rate dependence of the detection efficiency was also performed for a second detector with lower detection efficiency, see Figure 4 [Fig F0004b]. In order to do this, a detector of the same type (Perkin-Elmer-SPCM-AQR-16) as used in Section 4.9 was additionally equipped with a neutral density filter. This was just carried out in order to prove the validity of the simple model (Equation (4)) for any kind of intrinsic detection efficiency. Throughout the whole range of photon range impinging on the detectors, the ratio of the detection efficiencies can be modeled with the ratio derived from Equation (4).

**Figure 4a.**
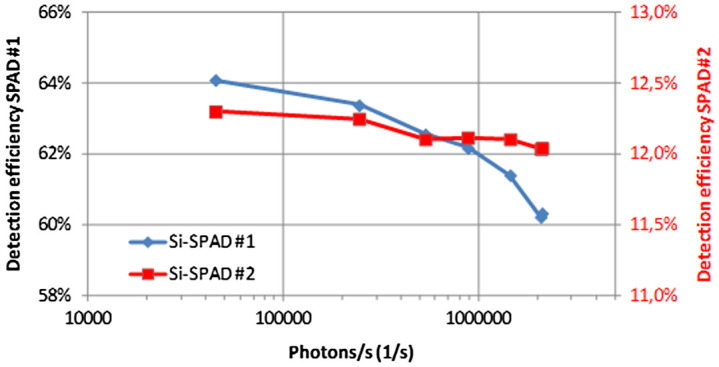
Detection efficiency of two Si-SPAD detectors of different intrinsic detection efficiency as a function of the photon rate. (The colour version of this figure is included in the online version of the journal.)

**Figure 4b. F0004b:**
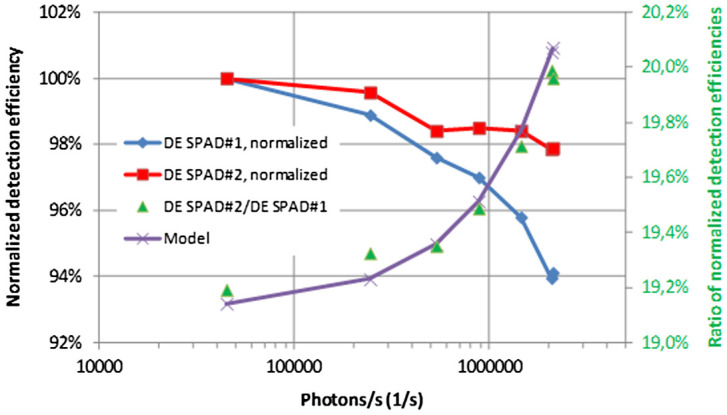
Normalized detection efficiency as a function of the photon rate. The photon rate dependence of the ratio of the detection efficiencies is also shown together with the model derived from Equation (4). (The colour version of this figure is included in the online version of the journal.)

## 5. Summary and outlook

In the paper, we have described a new setup for the determination of the detection efficiency of single-photon Si-photon avalanche diodes. The calibration process is based on the comparison with a standard Si-detector, traceable to the cryogenic radiometer, the primary standard for optical radiative power. The setup currently uses a tunable diode laser as radiation source; thus, the Poissonian statistics of the light has to be taken into account in the calibration process. The setup is in principle flexible for a broad wavelength range. A detailed uncertainty budget was presented and discussed. Currently, the expanded uncertainty (*k* = 2) obtained with the setup is very well below 1% for the detection efficiency. The main contributions for the uncertainty are the transmissions of the neutral density filters, which are necessary to adjust the photon flux rates to the detectors used in the calibration process. Moreover, the nonlinearity measurement of the standard detectors, although indicating a linear behavior, is a source of uncertainty, because the measurement of the filter transmission requires measurements covering a power level of 4–5 orders of magnitude. A dependence of detection efficiency from photon rate impinging on the detector is observed and can be described with a model, which accounts for photon statistics (Poissonian) and detector dead time.

Future work will focus on the improvement of the measurements of the filter transmission and detector nonlinearity, which should lead to a significantly reduced measurement uncertainty. Further issues to be tackled are the expansion of the wavelength range covered by the setup, the performance of international comparisons in order to validate the calibration results obtained and its associated uncertainties claimed, and the use of different kinds of light sources especially aiming towards the use of single-photon sources of high brilliance, which cover the detection ranges also of conventional detectors.
